# Nasal administration of anti-CD3 mAb (Foralumab) downregulates *NKG7* and increases *TGFB1* and *GIMAP7* expression in T cells in subjects with COVID-19

**DOI:** 10.1073/pnas.2220272120

**Published:** 2023-03-07

**Authors:** Thais G. Moreira, Christian D. Gauthier, Liam Murphy, Toby B. Lanser, Anu Paul, Kimble T. F. Matos, Davide Mangani, Saef Izzy, Rafael M. Rezende, Brian C. Healy, Clare M. Baecher-Allan, Tanuja Chitnis, Vijay Kuchroo, Howard L. Weiner

**Affiliations:** ^a^Department of Neurology, Ann Romney Center for Neurologic Diseases, Brigham and Women’s Hospital, Harvard Medical School, Boston, MA 02115; ^b^Escola Paulista de Medicina, Universidade Federal de São Paulo, Sāo Paulo 04023-900, Brazil

**Keywords:** Foralumab, T cells, COVID-19, anti-CD3, SARS-CoV-2

## Abstract

T cells are present in early stages of the severe acute respiratory syndrome coronavirus 2 (SARS-CoV-2) infection and play a major role in disease outcome and long-lasting immunity. Nasal administration of a fully human anti-CD3 monoclonal antibody (Foralumab) reduced lung inflammation as well as serum IL-6 and C-reactive protein in moderate cases of COVID-19. Using serum proteomics and RNA-sequencing, we investigated the immune changes in patients treated with nasal Foralumab. In a randomized trial, mild to moderate COVID-19 outpatients received nasal Foralumab (100 μg/d) given for 10 consecutive days and were compared to patients that did not receive Foralumab. We found that naïve-like T cells were increased in Foralumab-treated subjects and NGK7^+^ effector T cells were reduced. *CCL5,* *IL32,* *CST7,* *GZMH,* *GZMB,* *GZMA,* *PRF1*, and *CCL4* gene expression were downregulated in T cells and *CASP1* was downregulated in T cells, monocytes, and B cells in subjects treated with Foralumab. In addition to the downregulation of effector features, an increase in *TGFB1* gene expression in cell types with known effector function was observed in Foralumab-treated subjects. We also found increased expression of GTP-binding gene *GIMAP7* in subjects treated with Foralumab. Rho/ROCK1, a downstream pathway of GTPases signaling was downregulated in Foralumab-treated individuals. *TGFB1,* *GIMAP7*, and *NKG7* transcriptomic changes observed in Foralumab-treated COVID-19 subjects were also observed in healthy volunteers, MS subjects, and mice treated with nasal anti-CD3. Our findings demonstrate that nasal Foralumab modulates the inflammatory response in COVID-19 and provides a novel avenue to treat the disease.

## Significance

Activated T cells play an important role in the complications following COVID-19 infection. Anti-CD3 monoclonal antibody (mAb) binds to the T cell receptor and dampens inflammation by modulating T cell function. We show here that nasal administration of a fully human anti-CD3 Mab (Foralumab) modulates T cell inflammatory responses in COVID-19 by suppressing effector features in multiple T cell subsets, an effect also seen in subjects with multiple sclerosis. Immunomodulation by nasal anti-CD3 mAb represents a novel avenue for treatment of inflammatory human diseases.

The coronavirus disease 2019 (COVID-19) pandemic represents the greatest global public health crises since the pandemic influenza outbreak of 1918 (1). It is caused by the severe acute respiratory syndrome coronavirus 2 (SARS-CoV-2) that enters host cells through angiotensin-converting enzyme 2 (ACE2) and neuropilin (NRP1) receptors, resulting in a complex and heterogenous respiratory disease (2)*.* Although SARS-CoV-2 vaccination prevent hospitalization and mortality (3), concerns about newly emerging coronavirus variants that may be resistant to antibody-dependent vaccination strategies have spurred interest in other immune strategies to treat the disease (4, 5).

T cell responses are crucial for SARS-CoV-2 immunity. CD4^+^ and CD8^+^ SARS-CoV-2-specific T cells are present in early stages of the infection and increase over time. They have strong reactivity to the viral proteins and play a major role in lasting immunity (6–8). It has been shown that activated T cells increase in patients with moderate COVID-19 and do not return to normal levels during convalescence contributing to immunopathology which results in respiratory distress and organ damage (9)*.*

We and others have shown the modulation of T cell function by the oral or nasal administration of anti-CD3 monoclonal antibody in animal models of autoimmunity and inflammation including multiple sclerosis (MS) (10, 11), diabetes (12–14), arthritis (15)*,* inflammatory bowel diseases (16), and lupus (17). Anti-CD3 induces tolerogenic immune responses by stimulating T regulatory cells (18). In humans, we investigated the effects of nasal anti-CD3 in healthy volunteers and found that nasal administration of anti-CD3 monoclonal antibody (Foralumab) modulated effector CD8^+^ function and induced a regulatory transcriptional program in T cells (19).

We previously conducted a pilot trial administering nasal Foralumab in subjects with mild to moderate COVID-19 and found a reduction in serum IL-6 and C-reactive protein and more rapid clearance of lung infiltrates in treated individuals (1)*.* Here, we investigated the immune response of these subjects using whole-genome cell transcriptomics and serum proteomics and found that nasal Foralumab may dampen CD3^+^ T effector function by decreasing the GTPases of immunity-associated proteins (GIMAPs) associated pathway Rhoa/ROCK1 and *NKG7* gene expression while increasing *TGFB1* expression in COVID-19. We also show that this effect is not restricted to COVID-19 subjects but is also observed in healthy and multiple sclerosis (MS) subjects treated with nasal Foralumab. Taken together, we demonstrate a common mechanism of action of nasal anti-CD3 (Foralumab) that can be used therapeutically for subjects with COVID and other diseases with immune dysfuction.

## Results

### RNA Sequencing Identifies T Effector Subset Changes in Foralumab-Treated Subjects.

**Fig. 1.**
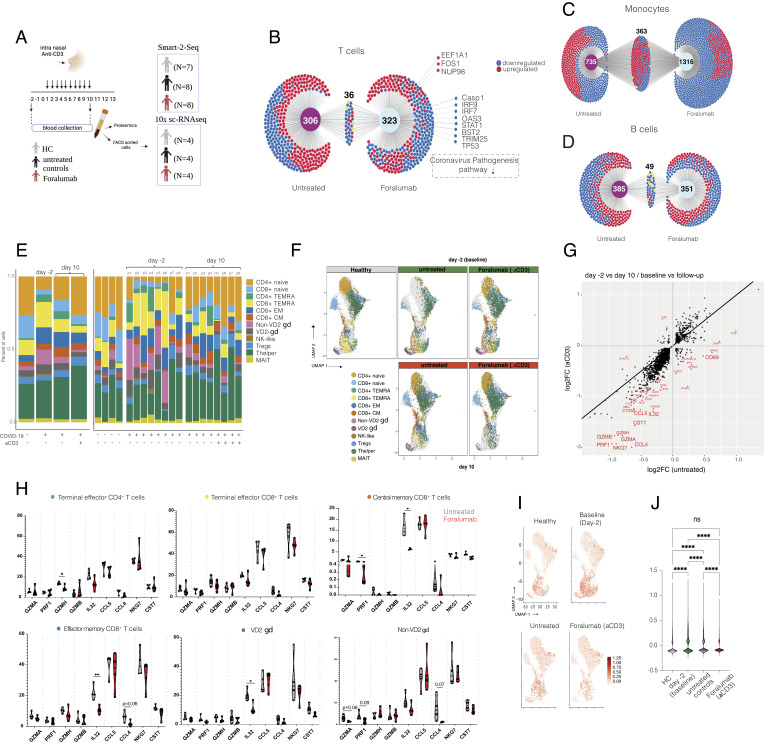
Compositional Analysis Shows Reduction of Effector T Cell Subsets in Foralumab-Treated subjects. Study design. Subjects were treated with 100 µg of nasal Foralumab given daily for 10 consecutive days. Blood was collected on day -2 and day 10. For bulk RNA-seq, CD3^+^, CD19^+^, and CD14^+^ cells were FACS-sorted from seven healthy volunteers, eight untreated controls, and eight Foralumab-treated subjects were studied (*B*-*D*). For 10× single cell RNA-seq CD3^+^ cells were FACS-sorted from four healthy volunteers, four untreated controls, and four Foralumab-treated subjects (*E*–*J*). (*B*-D**) DiVenn* Diagram shows differently expressed genes that were uniquely expressed in untreated subjects vs. Foralumab-treated subjects found in bulk-RNA-seq. Genes that were common between the groups are also shown. Red dots represent upregulated genes; blue dots represent downregulated genes. Differently expressed genes in T cell (*B*), monocytes (*C*) and B cells (*D*). (*E*) CD3+ cell subset distribution in healthy controls, untreated and Foralumab-treated COVID-19 subjects at baseline (day -2) and at day 10 identified by 10x sc RNA-seq. (*F*) Graph-based clustering of uniform manifold approximation and projection (UMAP) of T cell subsets at baseline (day -2) and at day 10 in healthy controls, untreated and Foralumab-treated COVID-19 subjects showing effector T cell cluster decrease in treated subjects. (*G*) Scatterplot of differentially expressed genes (DEGs) comparison between untreated controls and Foralumab-treated subjects at baseline (day-2) vs. follow-up (day 10). DEG lists from groups were crossed referenced and those above log2 fold change of 0.5 and below log2 fold change of −0.5 are labeled red. (*H*) Percentage of CD4^+^ and CD8^+^ TEMRAs, CD8^+^ CM, CD8^+^ EM, non-VD2 gamma delta (gd) and VD2 gd T cells expressing *GZMA,PRF1GZMH,GZMBI,L32,CCL5,CCL4,NKG7*, and *CST7*. Violin plots are median ± IQR. Student’s *t* test was used for comparison of untreated vs. Foralumab for each gene. (*I*) UMAP plots showing distribution of exhaustion-related genes *LAG3,TIGIT,PDCD1CTLA4,HAVCR2*, and *TOX*. (*J*) Exhaustion scores in each group.

To investigate the transcriptomic changes in immune cells following nasal Foralumab treatment in SARS-CoV-2-infected patients, we Fluorescence-activated cell sorting (FACS)sorted CD3^+^ (T cells), CD19^+^ (B cells), and CD14^+^ (monocytes) cells from Foralumab-treated and untreated COVID-19 patients at baseline (day -2) and day 10 as well as in healthy subjects (Fig. 1*A* and *SI Appendix*, Fig. S1 *A*, *B* and Table S1)(PRJNA899867 and PRJNA890877). Ingenuity pathway analysis (IPA) of the top differently expressed genes (DEGs) showed upregulation of coronavirus pathogenesis pathways in all three immune cell populations (*SI Appendix*, Fig. S1 *C*–*E*).

We then compared CD3^+^ cells isolated from COVID-19-infected subjects vs. healthy controls and found increased expression of activation markers including *CD69*, *CD83*, *ICOS*, *RGS1* as well as other stress related genes including *HIF1A*, *ATF4*, *NFKB1*, and *PR1*. Genes related to T cell responsiveness such as *IL21R*, *IL4R*, *CXCR4* were also upregulated in COVID-19-infected subjects (Dataset S1). Gene set enrichment analysis of DEGs showed upregulation of IL-18 (*P* < 0.001), TNF (*P* < 0.0001), and VEGFA-VEGFR2 signaling pathways (*P* = 0.002) in COVID-19 subjects (Dataset S2). In addition, activation of NLRP3 inflammasome by SARS-CoV-2 (*P* = 0.003) and host–pathogen interaction of human coronavirus interferon induction (*P* < 0.0001) canonical pathways was upregulated in T cells of COVID-19 subjects vs. healthy controls (*SI Appendix*, Fig. S1 *B*–*D* and Dataset S2). Overall, our findings are consistent with the known immune response in subjects infected with SARS-CoV-2 (20–23).

We next investigated the effect of nasal Foralumab on gene expression in T cells, B cells, and monocytes by comparing pre- treatment (day-2) vs. follow-up (day 10) in Foralumab-treated vs. untreated subjects. IPA of the differentially expressed genes (DEGs) before and after treatment showed downregulation of inflammatory pathways such as hypercytokinemia and interferon signaling pathway in both groups. We also identified a coronavirus pathogenesis pathway that were downregulated in the Foralumab group but not in untreated controls (Fig. 1*B* and Dataset S3). Of note, Caspase-1 was downregulated not only in T cells, but also in B cells and monocytes in Foralumab vs. untreated subjects (Dataset S4). Caspase-1 is associated with immune-related COVID-19 pathogenicity and worse outcome (24). In monocytes from Foralumab-treated individuals (Fig. 1*C*), we found that coronavirus pathogenesis pathway was slightly downregulated compared to untreated subjects (Dataset S3).

We thus analyzed the effect of nasal Foralumab on gene expression in B cells by comparing pre-treatment vs. day 10 in Foralumab-treated vs. untreated subjects Fig. 1*D* (Dataset S4). Although we found differences in gene expression in B cells, such as downregulation of IL-4 and IL-15 signaling in Foralumab-treated subjects, these differences were of smaller statistical significance compared to those observed in T cells (Dataset S3). Moreover, the in vivo effects of nasal Foralumab, that we observed on B cells and monocytes were indirect as B cells and monocytes do not express CD3 on their surface.

Our bulk RNA-seq analysis suggested that nasal administration of Foralumab led to the downregulation of genes related to inflammation and coronavirus pathogenesis and that its immunomodulatory effects were mostly pronounced in CD3+ T cells, although we could find some evidence for changes indirectly affecting both monocytes and B cells.

We also performed droplet-based 10x CellRanger sequencing in FACS sorted cells from healthy controls, untreated COVID-19 subjects, and Foralumab-treated COVID-19 subjects (Fig. 1*A*). We captured the transcriptomes of 12 cell types according to the expression of canonical gene markers (Fig. 1 *E* and *F* and *SI Appendix*, Fig. S2 *A*–*D* and Dataset S5). TCR sequencing on CD3^+^ cells (*SI Appendix*, Fig. S3 *A*–*D*) showed that the largest clonal expansion (clonal size 20 < X < 100) (*SI Appendix*, Fig. S3*C*), distributed in the lower regions of the UMAP and predominantly involved the CD8^+^ EM and TEMRA populations (*SI Appendix*, Fig. S3*D*). We found that Foralumab-treated subjects greatly recover from naïve CD4^+^ reduction observed at day -2 in COVID-19-infected subjects (*SI Appendix*, Fig. S2*D*). There were no differences in clonal expansion following Foralumab treatment.

We next compared gene expression of total CD3^+^ T cells in which we compared pre-treatment vs. day 10 for both Foralumab-treated subjects and untreated COVID-19 patients. We found significant downregulation of effector function genes including *NKG7*, *CCL5*, *IL32*, *CST7*, *GZMH*, *GZMB*, *GZMA*, *PRF1*, and *CCL4* in the Foralumab-treated subjects. (Fig. 1 *G* and *H* and Dataset S6 and *SI Appendix*, Fig. S2*E*). We further explored this effector gene distribution among the CD3+ subsets (Fig. 1*H*). The reduction of *IL32* was observed in CM and EM CD8^+^ cells as well as in gamma delta T cells in Foralumab-treated subjects. Downregulation of *GZMH* was observed in CD4^+^ effector cells and *PRF1* was downregulated in CD8^+^ CM (Fig. 1*H*). Taken together, our findings demonstrate a reduction of effector features in multiple T cell subsets in Foralumab-treated subjects when compared to untreated controls.

Because anti-CD3 activates T cells, we next investigated the cell state score of predefined gene sets associated to T cell exhaustion and activation in COVID-19 (9). By aggregating the expression values of coinhibitors molecules associated with T cell exhaustion, we were able to score and compare baseline (day -2) vs. follow-up (day 10) (Fig. 1 *I* and *J*). Foralumab-treated subjects showed greater reduction of co-inhibitors molecules associated with T cell exhaustion scores when compared to untreated controls. Of note, *MAF* and *TGFB1* gene expression was increased in highly exhausted cells when compared to non-exhausted cells while *IL2*, *TNFa* and *IFNg* was unchanged (*SI Appendix*, Fig. S4*A*, *B*). The coinhibitors molecules associated score was higher in Tregs, effector memory CD8+, CD8+ terminally differentiated effector memory (TEMRAS), and non-VD2 gamma delta T cells (*SI Appendix*, Fig. S4*C*).

### Changes in T Cell Expression of *GIMAP7*, *NKG7*, and *TGFB1* Are Associated with Immunomodulatory Effects of Nasal Foralumab.

We found that *NKG7* was downregulated in CD8^+^ TEMRAs, CD8^+^ EM, non-VD2 gamma deltas, and gamma delta T cells when compared to baseline (day -2) in COVID-19-treated subjects but not untreated controls (*SI Appendix*, Fig. S5*A*). *TGFB1* was increased in multiple CD3^+^ subsets including CD4^+^ TEMRA, CD8^+^ TEMRA, CD8^+^ EM, non-VD2 gamma-delta, and VD2 gamma-delta T cells following Foralumab treatment when compared to both healthy controls and untreated COVID-19 subjects. The *TGFB1* gene expression increase was found in cell types with known effector function that not classical Treg cells (*SI Appendix*, Fig. S5*B*).

**Fig. 2.**
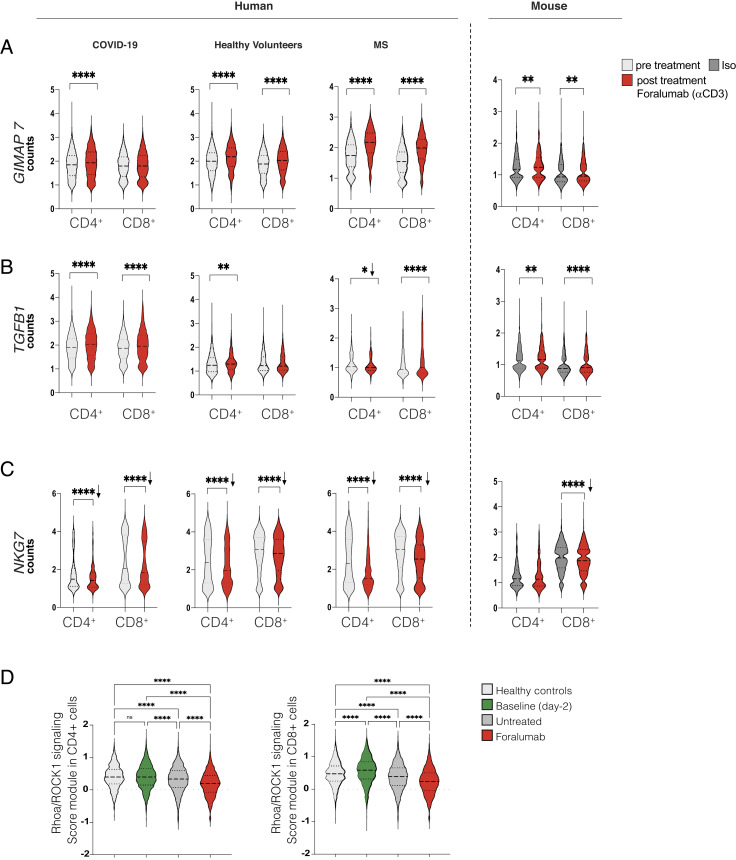
*GIMAP7, TGFB1*, and *NKG7* Gene Expression Changes Play a Key Role in Foralumab Modulatory Effects. (*A*–*C*) Foralumab-modulated gene expression of *GIMAP7* (*A*) *TGFB1* (*B*) and *NKG7* (*C*) counts across cell types and treatment. COVID-19 subjects were treated intranasally with 100 µg of Foralumab for 10 consecutive days. Analysis shows before treatment (day -2) and after Foralumab (day 10). Data from healthy volunteers were obtained in a dose escalation study. Selected data reflect a dose of 50 µg given for 5 d (n = 6). Subjects were followed up for 30 d. MS subject is a 61-y-old male with non-active progressive MS treated with 50 µg of Foralumab three times a week for two consecutive weeks (one cycle) followed by 1 wk interval. A total of five cycles was completed. Analysis shows before treatment vs. after treatment. C57BL/6 mice were treated intranasally with 1 µg of anti-CD3 (clone 145-2C11, Novus Biologicals) or isotype control (Iso) for five consecutive days. CD3^+^ cells from cervical lymph node were FACS-sorted. For human studies, CD3+ cells were obtained from PBMC. Violin plots are median ± IQR. Student’s *t* test. *P < 0.05, **P < 0.01, ***P < 0.001, ****P < 0.001. Arrows shows downregulation. (*D*) Gene expression levels of *RhoAROCK1and CFL1* values was aggregated (RhoA/ROCK1 pathway) and scored in CD4+ and CD8+ cells of healthy controls, Baseline (day -2) and untreated and Forlaumab subjects on day 10. One-way ANOVA, followed by Tukey post hoc was used for analysis. ****P < 0.001

In addition to COVID-19 subjects, we have treated healthy volunteers (19) and multiple sclerosis (MS) subjects (25) with nasal Foralumab and performed scRNA-seq analysis on CD3+ subsets in these subjects. This provided the opportunity to ask whether there were common mechanisms by which nasal anti-CD3 modulated T cell function in different human conditions. We found that *GIMAP7* and *TGFB1* gene expression were upregulated, whereas *NKG7* gene expression was downregulated in all three cohorts (Fig. 2 *A*–*C*). Furthermore, we found these same changes in CD3+ cells sorted from the cervical lymph nodes of C57BL/6J mice treated with nasal anti-CD3 (Fig. 2 *A–C*). Although the immunologic role of GIMAPs is not well defined, they define stages of T cell development, survival, and cell identity (26, 27) and have been reported to be associated with T cell regulatory function (28)*.*

Lastly, we found that the gene expression of *RhoA*, *ROCK1, and CFL1*, which are negatively regulated by GTPases was downregulated in Foralumab-treated individuals. By aggregating the expression values of these genes, we were able to score the changes in the Rhoa/ROCK1 pathway and found a decrease of this pathway in Foralumab-treated subjects (Fig. 2*D*).

Additionally, we quantified serum cytokines, growth factors and chemokines in COVID-19 subjects following Foralumab treatment as well as in untreated COVID-19 and healthy controls using both OLINK and Multiplex technology (*SI Appendix*, Fig. S6 *A* and *B*). We found an increase in brain-derived neurotrophic factor (BDNF) and growth factors in Foralumab treated patients that was not observed in untreated COVID-19 subjects or healthy controls (*SI Appendix*, Fig. S7*A* and Table S2 and Dataset S7). Increased BDNF secretion has been associated with recovery in COVID-19 (29). Two serum proteins (FLT3L and IL-12B), were reduced by Foralumab treatment and four proteins were increased following treatment (NT-3, ST1A1, AXIN1 and SIRT2). Of note, neurotrophin-3 (NT-3) is a member of the BDNF family that we also found increased in Foralumab-treated subjects. SIRT2 plays a role in T cell metabolism and effector function (30)*.* (*SI Appendix*, Fig S7*B* and Dataset S8). Although the neuronal growth factor BDNF is produced by immune cells including T cells (31), we did not find BDNF expression in CD3+ subsets. We found that FLT3L, SIRT2, SULT1A1, and AXIN1 genes were expressed in T cells in both untreated and Foralumab treatment CD3+ subsets (*SI Appendix*, Fig S7 *C* and *D* and Dataset S8).

Taken together, we found that nasal anti-CD3 (Foralumab) induces the secretion of factors related to tissue remodeling, induces naïve-like cells and restrains effector features in CD3^+^ T cells as exemplified by decreased gene expression of *CASP1*, *CCL4*, and *NKG7*, while *TGFB1* gene expression is increased. Notably, the transcriptome changes found in CD3+ subsets upon Foralumab treatment are not limited to subjects with COVID-19 but was also observed in healthy and MS subjects treated with nasal Foralumab.

## Discussion

T cells play an important role in subjects with COVID-19 infection as COVID-19 patients develop hyperactive T cell responses that contribute to disease pathogenesis (6). Thus, in addition to vaccination and anti-viral strategies to combat COVID-19, dampening of hyperactive T cell responses is a goal of COVID-19 therapeutics (4)*.* We previously reported positive effects of nasal anti-CD3 (Foralumab) by reducing inflammatory markers and lung infiltration in subjects with mild to moderate COVID-19 (1)*.* In the present study, we performed detailed immunological analysis to understand how nasal Foralumab-modulated T cell function in COVID-19-treated patients.

We found that *NKG7*, *IL32*, *GZMA*, *PRF1*, *CST7*, *GZMH*, and *CCL5* were among the top DEG downregulated in CD3^+^ upon nasal Foralumab treatment of COVID-19 subjects. In addition to decreased expression of genes related to effector function in T cells, we further found an increase of *TGFB1* expression in effector COVID-19 patients treated with nasal Foralumab. *TGFB1* expression was found to be increased in effector cells but not naïve or Treg cells suggesting that Foralumab acted to restrain T cell effector function rather than inducing Treg cells in the periphery. A decrease in CD8+ effector function was also observed in subjects with diabetes treated intravenously with anti-CD3 (Teplizumab) (32). In vitro effects of Foralumab demonstrated that Foralumab-stimulated CD8+ T cells exhibited regulatory function via their capacity to kill CD4+ T cells and that LAP (TGFβ), TIGIT, and KLRG1 were induced on in vitro Foralumab-stimulated CD8+ T cells (19)*.*

In addition to treatment of COVID-19 subjects with nasal Foralumab, we have treated healthy volunteers (19) and subjects with progressive MS with nasal Foralumab (25)*.* This provided the opportunity to determine whether there were common changes in human subjects treated with nasal Foralumab-treated subjects. Indeed, we found that nasal Foralumab decreased *NKG7* and *GIMAP7* and increased *TGFB1* mRNA expression. Of note, these changes were also observed in the cervical lymph nodes of mice treated with nasal anti-CD3.

We chose a dose of 100 μg for 10 d in COVID-19 patients based on results in healthy volunteers and dose-response curves in animals given nasal anti-CD3. What is remarkable in our findings is that identical gene changes were observed in healthy subjects given 50 μg × 5 d, COVID-19 subjects given 100 μg × 10 d, as well as in MS subjects given 50 μg in an every-other-day regimen at 2-wk intervals. We believe this adds to the robustness of our findings and demonstrates that the dose used (50 to 100 μg) is in the appropriate range to induce immune effects of nasal Foralumab. In addition, identical gene expression changes were observed in animals treated with nasal anti-CD3 at a dose of 1 μg for 5 d. Formal clinical trials will be needed to establish an optimal dosing regimen.

We propose a mechanism of action whereby nasal Foralumab in humans involves the induction of a quiescence program in T cells through the upregulation of *GIMAP7* and *TGFB1*, concomitantly with the suppression of the *NKG7* related genes, including *PRF1* and *GMZ*. The interactions among GIMAPs, NKG7, and TGFβ1 to affect immune activation has not been described before and may represent an important pathway to maintain immune homeostasis. Of note, the *CASP1* gene that was decreased following nasal Foralumab and the *CD3E* gene that is targeted by Foralumab are coexpressed with *GIMAP7*, which we found to be increased at the gene expression level.

We also found that the Rhoa/ROCK1 pathway, known to be negatively regulated by GTPases was downregulated in Foralumab-treated subjects. Several important immunomodulatory roles of the Rhoa/ROCK1 signaling pathway and its respective genes in T cell function have been shown including the role of Cofilin in T cell hyporesponsiveness (33) and increased ROCK activity in dysfunctional T cells (34, 35). Particularly to the CNS, Rho/ROCK signaling pathway in T cells was shown to inhibit regeneration following CNS damage (36)*.* Moreover, reduced EAE severity was shown in mice with a T cell-specific deletion of RhoA gene (37). Thus, our data suggest an inhibition of the Rho/ROCK1 signaling pathway as a downstream effect of *GIMAP7* upregulation in Foralumab-treated subjects that may play a role in Foralumab immunomodulatory effects including its neuroprotective role.

BDNF levels were increased in the overall COVID-19 cohort at baseline and several subjects in the subsequent non-treatment cohort had high baseline values. It has been shown that COVID-19 may be associated with increased BDNF perhaps related to an immune response to modulate the disease (29, 38). We found that nasal Foralumab increased in BDNF levels, which is a potential mechanism by which it may affect the disease. Low levels of BDNF are associated with a worse prognosis (29). BDNF can be produced by several cell types including leukocytes, neurons, and adipose tissue. We did not observe significant BDNF gene expression in CD3+ cells.

In summary, we found that nasal Foralumab modulates T cell inflammatory responses that occurs in SARS-CoV-2 infection by suppressing effector features in multiple CD3^+^ T cell subsets.

Although our cohort it is limited by its size and is not powered to present a biomarker associated with clinical outcomes in COVID-19, the major finding in our study is the identification of a unique mechanism associated with nasal Foralumab in humans that will help advance use of Foralumab as an adjunct therapy for COVID-19 and guide long-term use of this therapy in autoimmune conditions.

## Material and Methods

### Study Design and Subject Groups.

Patients with mild to moderate COVID-19 were recruited and treated at the Santa Casa de Misericordia de Santos in São Paulo State, Brazil, as previously described (1). Subjects were treated with 100 μg nasal Foralumab given daily for 10 consecutive days and blood used for immunologic analysis was collected at baseline (day -2) and day 10 (Fig. 1*A*). We administrated nasal Foralumab to healthy subjects at doses of 10 μg, 50 μg, and 250 μg given for five consecutive days and the immune effects were predominantly observed at the 50 μg dose (19). We chose a dose of 100 μg for 10 d in COVID-19 patients based on these results in healthy volunteers and dose-response curves in animals given nasal anti-CD3. Blood samples were collected under ethical committee approval from the Universidade Metropolitana de Santos UNIMES (CAAE: 38056120.1.0000.5509). Immunologic studies were performed at the Brigham and Women’s Hospital, Boston, under Institutional Review Boards (IRB) approval 2020P000563. All studies performed at the Brigham and Women’s Hospital were approved by the Mass General Brigham Human Subjects Research Committee (IRB). All participants voluntarily signed a consent form agreeing with the procedures and were informed that immunologic studies were being performed on their blood samples. Of the 12 Foralumab-treated subjects and 16 untreated subjects in the trial, 8 from each group were used for immunologic analysis. For bulk RNAseq, eight Foralumab-treated, eight untreated subjects, and seven healthy controls were studied. For scRNA-seq, four subjects from each group were studied. For OLINK and multiplex analysis, 12 Foralumab-treated subjects, 15 untreated subjects, and 6 healthy controls were used (*SI Appendix*, Table S1). Foralumab (28F11-AE; NI-0401) is a fully human IgG1 anti-CD3 mAb with the Fc portion mutated such that the mAb is non-FcR binding in vitro which exhibits only minor cytokine release in vivo while maintaining modulation of the CD3/TCR and T cell depletion (39, 40) Foralumab was developed by Novimmune and was acquired by Tiziana Life Sciences.

### PBMC Isolation.

Human peripheral blood mononuclear cells (PBMC) isolation was performed within 4 h after blood collection according with standard protocol (41). Briefly, centrifuged cells were mixed with Ficoll solution. Plasma layer was removed and storage. PBMC pellet was washed with Hanks’ Balanced Salt Solution (HBSS) solution and then resuspended in freezing medium (10% DMSO, 50% FBS, 40% RPMI) and transferred to cryovials. Slow freezing technique using Mr. Frosty Container was used. Cryovials were long-term storage in liquid nitrogen. Aliquots of serum were also obtained and were frozen for later analysis. Samples were shipped in dry ice to The Mass General Brigham, Boston, USA.

### Flow Cytometry and Cell Sorting.

PBMCs were thawed at 37C into complete RPMI media with 5% human AB serum (Gemini Bioproducts) in the presence of benzonase [20 mL/10mL], washed with PBS and resuspended in Fcr Block human TruStain FcX™ (1:20) and stained for viability (eFluor 506 viability dye, Invitrogen, 1:1,000). 3 × 10^6^ cells from each sample were stained with anti-human CD3 (APC-Cy7, SK7, 1:300, Biolegend), anti-CD19 (APC, 4G7, 1:300, Biolegend), anti-CD14 (FITC, M5E2, 1:200, BD pharmigen) and anti-CD66b (Pe, G10F5, 1:300, BD Pharmigen). Cell sorting was performed using BD FACS ARIA.

### Single-Cell 5’mRNA Sequencing and Bulk Smart-seq2 RNA-Seq.

Immune cells from participants that received Foralumab and untreated controls without the use of dexamethasone (*SI Appendix*, Table S1) as well as uninfected patients used as healthy controls were analyzed by scRNA-seq using the 10X Genomics platform. CD3+ T cells were FACS-sorted from baseline (day-2) and day 10 samples and used for single-cell sequencing. Additionally, T cell, B cells, and monocytes were FACS sorted from similar FACS sorted gating setting and processed for bulk Smart-seq2-RNAseq. Single-cell RNA-seq experiments were performed by the Brigham and Women’s Hospital Single-cell Genomics Core. Sorted cells were stained with a distinct barcoded antibody (Cell-Hashing antibody, TotalSeq-C, Biolegend) as previously described (42). Next, cells from each condition were pooled together and resuspended in 0.4% BSA in PBS at a concentration of 2,000 cells per μL, then loaded onto a single lane (Chromium chip K, 10X Genomics) followed by encapsulation in a lipid droplet (Single-cell 5′kit V2, 10X Genomics) followed by cDNA and library generation according to the manufacturer’s protocol. 5′ mRNA library was sequenced to an average of 50,000 reads per cell, V(D)J library and HTO (Cell Hashing antibodies) library sequenced to an average of 5,000 reads per cell, all using Illumina Novaseq. scRNA-seq reads were processed with Cell Ranger, which demultiplexed cells from different samples and quantified transcript counts per putative cell. The quantification was performed using the STAR aligner against the GRCh38 transcriptome. Smart-seq2 RNA-seq was performed by the Broad Genomics platform, Broad Institute of MIT and Harvard. Cells were collected in RNA-free microtubes containing 5 μL of TCL buffer Qiagen, (1031576) and 1% 2-mercaptoethanol (Gibco). Lysate cleanup and reverse transcription of mRNA, transcriptome amplification and PCR cleanup, Nextera XT sequencing-library construction, DNA SPRI bead cleanup and 2 × 38 bp paired sequencing were performed. The sequencing was run on all Illumina NextSeq500. T cell receptor (TCR) repertoire analysis was conducted primarily through the scRepertoire (v1.3.5) package in R. The filtered contig annotation outputs from CellRanger were preprocessed and mapped to individual cell barcodes using the *createHTOContigList()* and *combineTCR()* functions. Resultant demultiplexed TCR data were then merged with the single-cell objects created with Seurat (v.4.1.1) prior to analysis and visualization. All plots were generated using tools within scRepertoire, ggplot2 (v3.3.6), and dittoSeq (v1.4.4).

### Sequencing Analysis.

Venn diagram visualizations were created using Integrated web-based visualization tool (DiVenn) used for Bulk-RNA-seq visualization (43)**.** Bulk RNA sequencing samples were processed according to the Smart-Seq2 protocol and sequenced on Illumina sequencers. Reads in FASTQ were quantified at the transcript level using Salmon (v1.4.0) against the H. Sapiens Ensembl gene catalog (August 2020). Differential expression analysis was conducted using R (v4.0.3) and DESeq2 (v1.30.1). Hypothesis testing was performed by using either DESeq2’s Wald test on appropriate variable contrasts or with a Likelihood Ratio Test (LRT) for models with greater than two variable conditions being tested. For scRNA-seq, the Seurat package was used to process and analyze the data using default parameters. Harmony was used to batch correction and Celldex was used for cell type annotation. After the unified single-cell analysis, pipeline unique transcripts were obtained from 22,000 singlet cells from all samples. All high-quality cells were integrated into an unbatched and comparable dataset and subjected to principal component analysis after correction for read depth and mitochondrial read counts. The AddModuleScore function in Seurat was used to implement the method with default settings found in (9). Exhaustion markers were (*LAG3, TIGIT, PDCD1, CTLA4, HAVCR2*, and *TOX*). Coronavirus pathway genes that were interfaced in the single-cell data are *CASP1, IRF9, OAS3, STAT1, BST2, FOS, IRF7, NUP98, STAT2*.

### Olink Proteomics and Multiplex.

Inflammation panel comprising of 96 protein biomarkers (Olink Proteomics) was used on serum samples of patients at baseline (day-2) and day 10. Data from the analyzed protein biomarkers was presented in normalized protein expression (NPX) values, Olink Proteomics’s arbitrary unit on log2 scale. Cytokine/Chemokine/Growth Factor Panel (EPX450-12171-901) in serum samples in patients treated with Foralumab, untreated control, and healthy controls. Assay was performed by The Cytokine Core, LLC according to the manufacturer’s instructions. Markers were BDNF, EGF, Eotaxin, FGF-2, GM-CSF, GRO-α*, HGF, IFN-α, IFN-γ, IL-1RA, IL-1α, IL-1B, IL-2, IL-4, IL-5, IL-6, IL-7, IL-8, IL-9, IL-10, IL-12(p70), IL-13, IL-15, IL-17A, IL-18, IL-21, IL-22, IL-23, IL-27, IL-31, IP-10, LIF,MCP-1, MIP-1α, MIP-1β, NGF-β, RANTES, PDGF-BB, PIGF-1, SCF, SDF-1α, TNF-α, TNF-β, VEGF-A, VEGF.

### Statistical Analysis.

Results are shown as the mean values (±SEM) or median (±IQR) and considered statistically significant when comparisons between groups, using Student’s *t* test, one-way or two-way ANOVA *P* values were less than 0.05. *P < 0.05 *P* < 0.05 **P < 0.01***P < 0.001 ****P < 0.0001. Changes from Baseline was used for Olink and Multiplex analysis.

## Supplementary Material

Appendix 01 (PDF)

Dataset S01 (XLSX)

Dataset S02 (XLSX)

Dataset S03 (XLSX)

Dataset S04 (XLSX)

Dataset S05 (XLSX)

Dataset S06 (XLSX)

Dataset S07 (XLSX)

Dataset S08 (XLSX)

## Data Availability

RNA-seq and single cell raw data files can be found in NCBI platform PRJNA899867 and PRJNA890877. https://dataview.ncbi.nlm.nih.gov/object/PRJNA899867?reviewer=cc8a4hnclmk 96f46ihb0fotdgv and https://dataview.ncbi.nlm.nih.gov/object/PRJNA890877?reviewer= d2pvv5i20kltf0p86igs962ish.
