# Supplementary material for: Nasal administration of anti-CD3 mAb (Foralumab) downregulates NKG7 and increases TGFB1 and GIMAP7 expression in T cells in subjects with COVID-19
Source: Proc Natl Acad Sci U S A. 2023 Mar 7;120(11):e2220272120. doi: 10.1073/pnas.2220272120 (PMC10243127; doi:10.1073/pnas.2220272120)
Supplement: Supplementary file 1 — Appendix 01 (PDF) [file pnas.2220272120.sapp.pdf]

Supporting Information for

Nasal administration of anti-CD3 mAb (Foralumab) downregulates *NKG7* and increases *TGFb1* and *GIMAP7* expression in T cells in subjects with COVID-19.

Thais G. Moreira[1], Christian D. Gauthier[1], Liam Murphy[1], Toby B. Lanser[1], Anu Paul[1], Kimble T. F. Matos[2], Davide Mangani[1], Saef Izzy[1], Rafael M. Rezende[1], Brian C. Healy[1], Clare M. Baecher-Allan[1], Tanuja Chitnis[1], Vijay Kuchroo[1], Howard L. Weiner [1]

Correspondence: hweiner@rics.bwh.harvard.edu / tmoreira@bwh.harvard.edu/
* Howard L. Weiner / Thais Garcias Moreira
Ann Romney Center for Neurologic Diseases
Department of Neurology,
Brigham and Women's Hospital,
Harvard Medical School, Boston, MA, USA.
60 Fenwood Rd, Boston, MA. 02115 USA

This PDF file includes:

Figures S1 to S7
Tables S1 and S2

Other supporting materials for this manuscript include the following:

Datasets S1 to S8

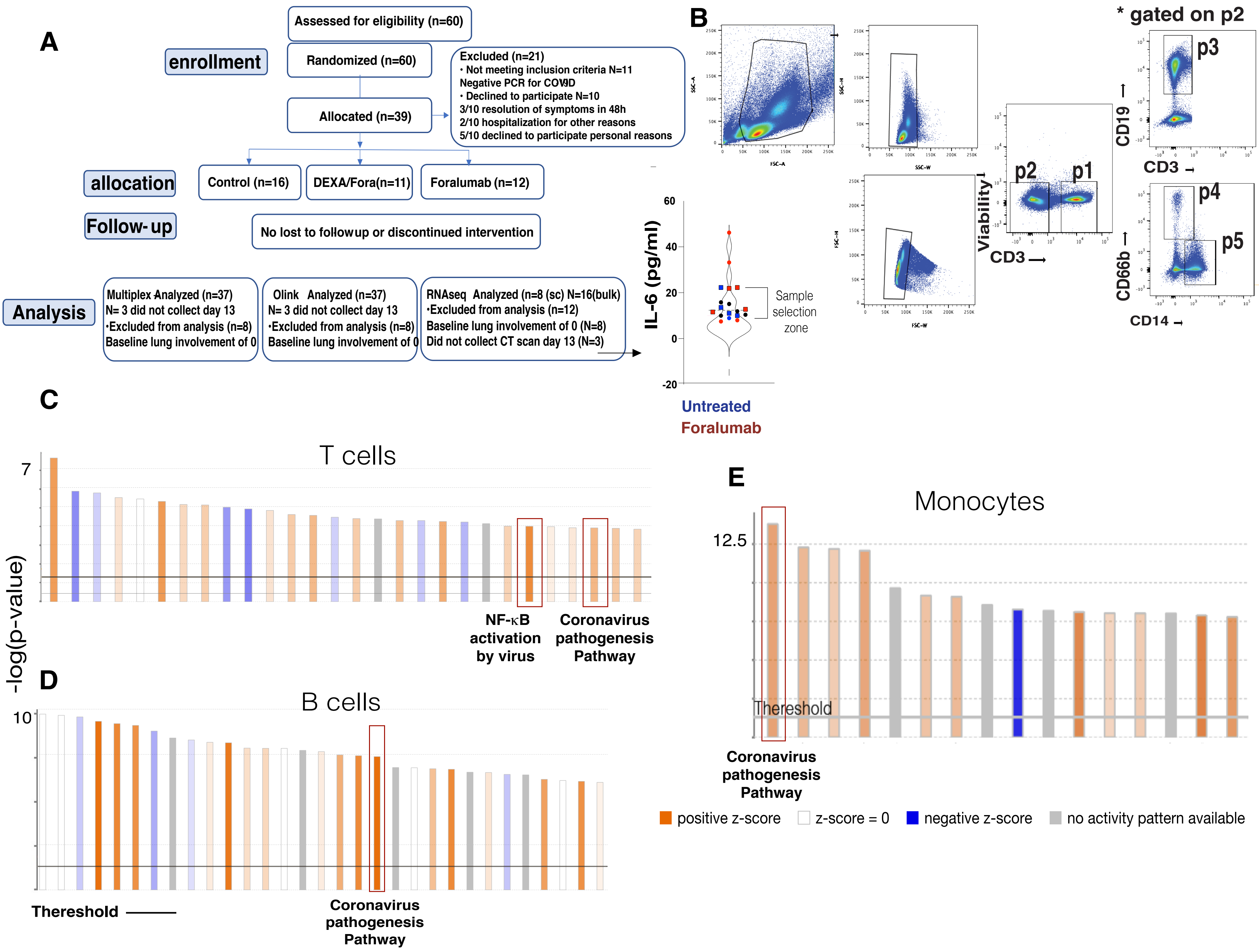

A
enrollment
Assessed for eligibility (n=60)
Randomized (n=60)
Allocated (n=39)
Excluded (n=21)
• Not meeting inclusion criteria N=11
Negative PCR for COVID
• Declined to participate N=10
3/10 resolution of symptoms in 48h
2/10 hospitalization for other reasons
5/10 declined to participate personal reasons
allocation
Control (n=16)
DEXA/Fora(n=11)
Foralumab (n=12)
Follow-up
No lost to followup or discontinued intervention
Analysis
Multiplex Analyzed (n=37)
N= 3 did not collect day 13
•Excluded from analysis (n=8)
Baseline lung involvement of 0
Olink Analyzed (n=37)
N= 3 did not collect day 13
•Excluded from analysis (n=8)
Baseline lung involvement of 0
RNAseq Analyzed (n=8 (sc) N=16(bulk)
•Excluded from analysis (n=12)
Baseline lung involvement of 0 (N=8)
Did not collect CT scan day 13 (N=3)
B
IL-6 (pg/ml)
Sample selection zone
Untreated
Foralumab
* gated on p2
p1
p2
p3
p4
p5
Viability
CD3
CD19
CD66b
CD14
C
T cells
7
-log(p-value)
NF-κB activation by virus
Coronavirus pathogenesis Pathway
D
B cells
10
Thereshold
Coronavirus pathogenesis Pathway
E
Monocytes
12.5
Thereshold
Coronavirus pathogenesis Pathway
positive z-score
z-score = 0
negative z-score
no activity pattern available

Figure S1. Gating strategy for RNA-sequencing and Ingenuity Pathway Analysis A) Sample selection for RNA-sequencing. 39/60 patients were allocated. Samples from Fora/Dexa group were not elected for this study and thus 28/39 patients were selected. Further, 12/28 samples were removed because patients did present lung involvement or did not collect CT scan on endpoint and thus could not be evaluated. We then used IL-6 serum levels at baseline to define a sample selection zone that excluded skewed IL-6 serum levels. Samples within median and interquartile range were used for single cell RNA-seq analysis and it is shown in squared shape. Bulk RNA-seq **B)** Gating strategy for cell sorting: bulk RNA-seq (Smart-Seq2) and 10x single cell RNA-seq**.** PBMCs were stained with viability dye, CD3, CD19, CD66b and CD14 and FACS-sorted. Cells were gated on singlet+ Live+ cells. P1 ($CD3^+$), P3 ($CD19^+$) and P5 ($CD14^+$) were gated on P2, $CD3^-$. **C-E)** Ingenuity Pathways Analysis on DEG genes ($p<0.05$) of comparisons between healthy volunteers and COVID-19 subjects in $CD3^+$ T cells, $CD19^+$ B cells and CD14+ monocytes . N=7 healthy volunteers (HV), N=8 untreated controls and N=8 Foralumab treated

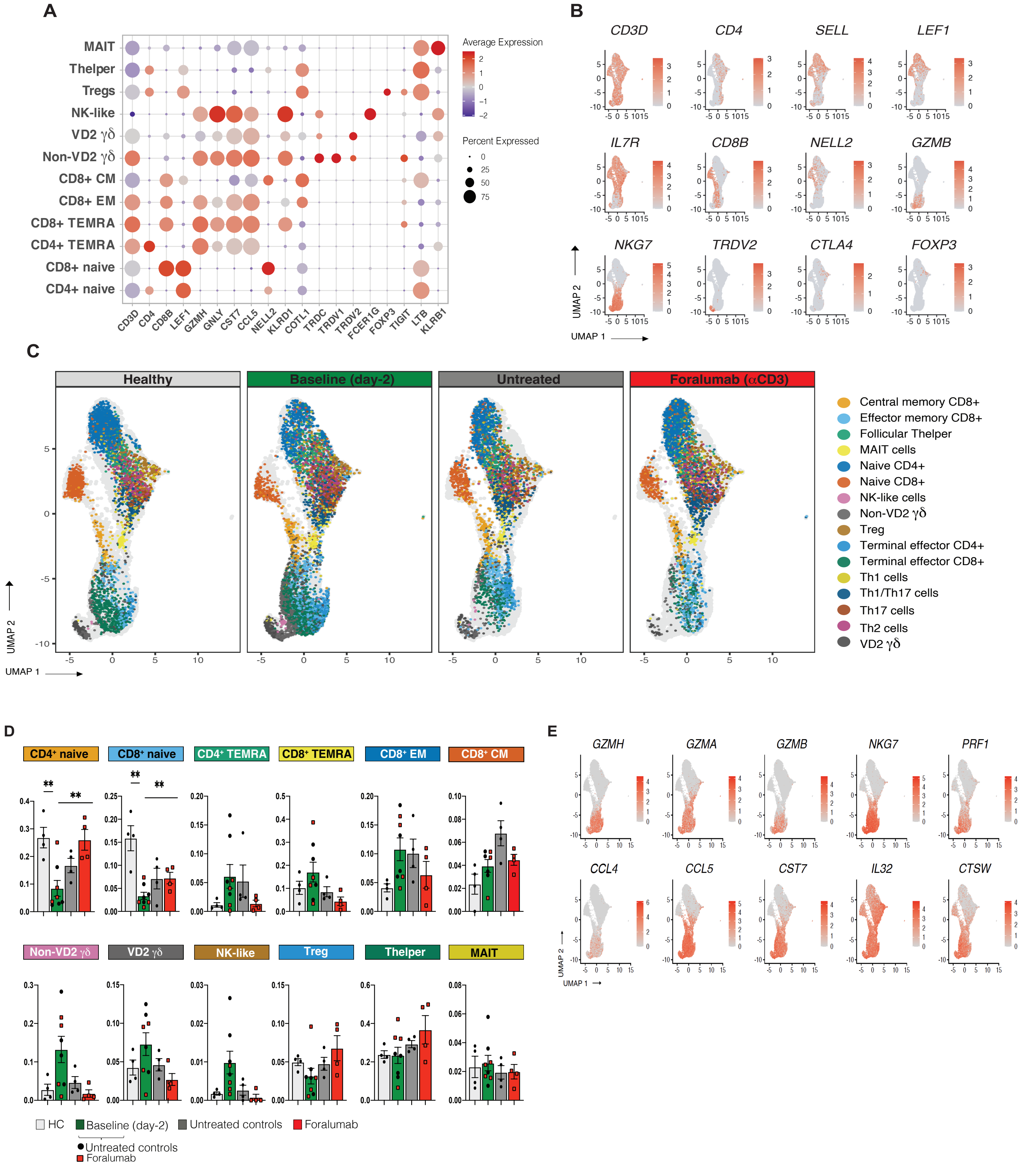

A
MAIT
Thelper
Tregs
NK-like
VD2 γδ
Non-VD2 γδ
CD8+ CM
CD8+ EM
CD8+ TEMRA
CD4+ TEMRA
CD8+ naive
CD4+ naive
CD3D CD4 CD8B LEF1 GZMH GNLY CST7 CCL5 NELL2 KLRD1 COTL1 TRDC TRDV1 TRDV2 FCER1G FOXP3 TIGIT LTB KLRB1
Average Expression
Percent Expressed
B
CD3D CD4 SELL LEF1
IL7R CD8B NELL2 GZMB
NKG7 TRDV2 CTLA4 FOXP3
UMAP 2
UMAP 1
C
Healthy
Baseline (day-2)
Untreated
Foralumab (αCD3)
Central memory CD8+
Effector memory CD8+
Follicular Thelper
MAIT cells
Naive CD4+
Naive CD8+
NK-like cells
Non-VD2 γδ
Treg
Terminal effector CD4+
Terminal effector CD8+
Th1 cells
Th1/Th17 cells
Th17 cells
Th2 cells
VD2 γδ
D
CD4+ naive
CD8+ naive
CD4+ TEMRA
CD8+ TEMRA
CD8+ EM
CD8+ CM
Non-VD2 γδ
VD2 γδ
NK-like
Treg
Thelper
MAIT
HC
Baseline (day-2)
Untreated controls
Foralumab
Untreated controls
Foralumab
E
GZMH GZMA GZMB NKG7 PRF1
CCL4 CCL5 CST7 IL32 CTSW
UMAP 2
UMAP 1

Figure S2. T cell subtyping. A) Canonical cell markers for T cell subsets. **B)** UMAP plots of T cells showing localization of $CD3^+$ cell subset markers. **C)** CD3+ subsets including T helper stratification into Th1, Th2 and Th17 subsets. **D)** Graphic representation T cell subset distribution in healthy controls, untreated and Foralumab treated COVID-19 subjects at baseline (day-2) and at day 10 shown in Fig1E. Bars represent mean$\pm$SEM. One-way ANOVA followed by Tukey *post hoc* analysis ** p<0.01. **E)** UMAP plots of T cells showing localization of effector genes.

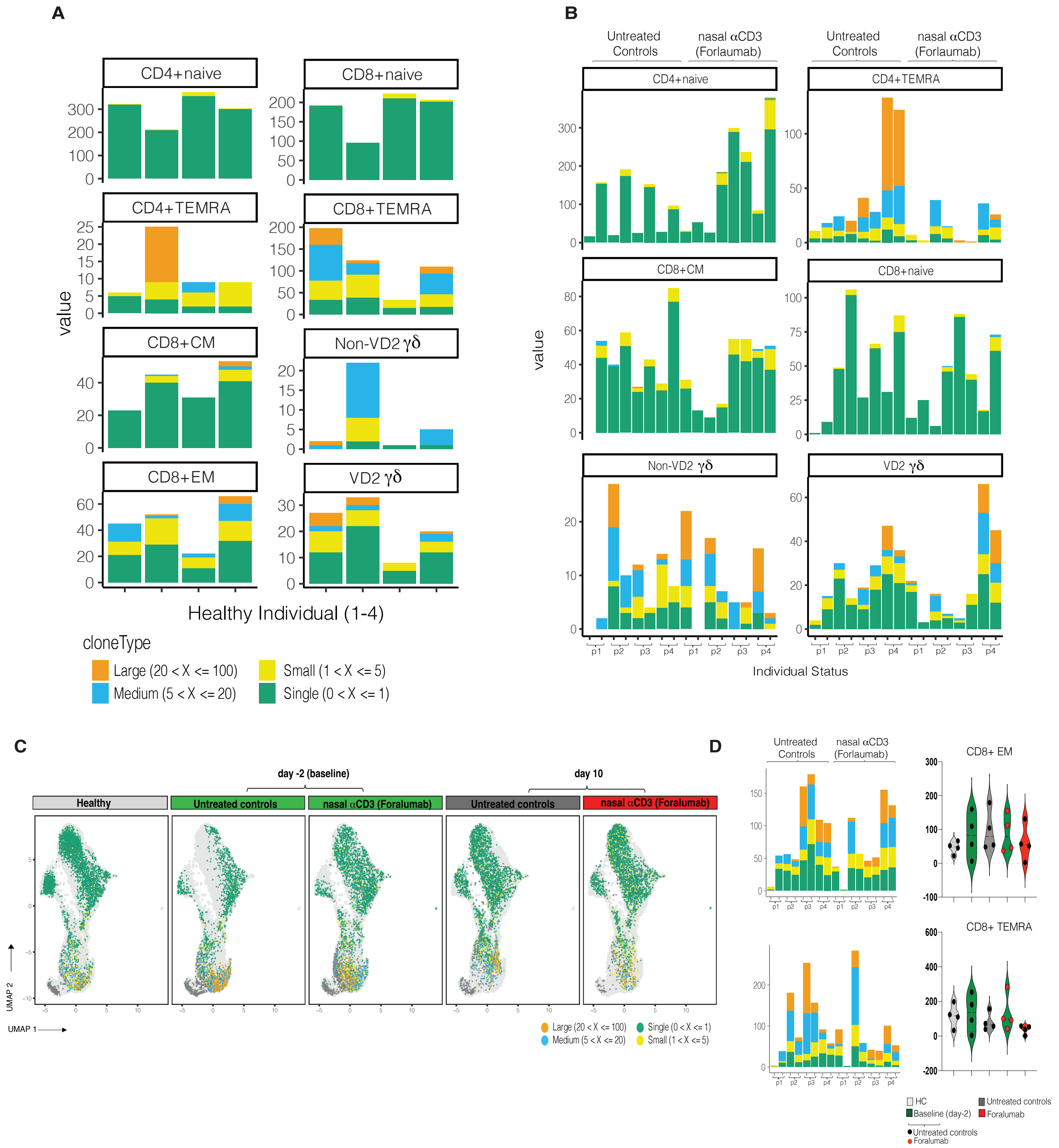

Figure S3. TCR sequencing in Foralumab treated subjects. A, B) TCR sharing patterns of specific T cell subsets by comparing usage of V(D)J genes on healthy controls **(A)**, untreated controls vs. Foralumab treated subjects **(B)**. **C)** Distribution within UMAP plots of T cells subsets. **D)** Violin plots showing the frequency of clonal T cells in CD8^{+} TEMRA and CD8^{+} CM cells (Median$\pm$IQR). Graphs show percentage of each clonal type and individual status. 4 healthy volunteers, 4 untreated controls (before and after) and 4 Foralumab treated subjects (before and after) were studied.

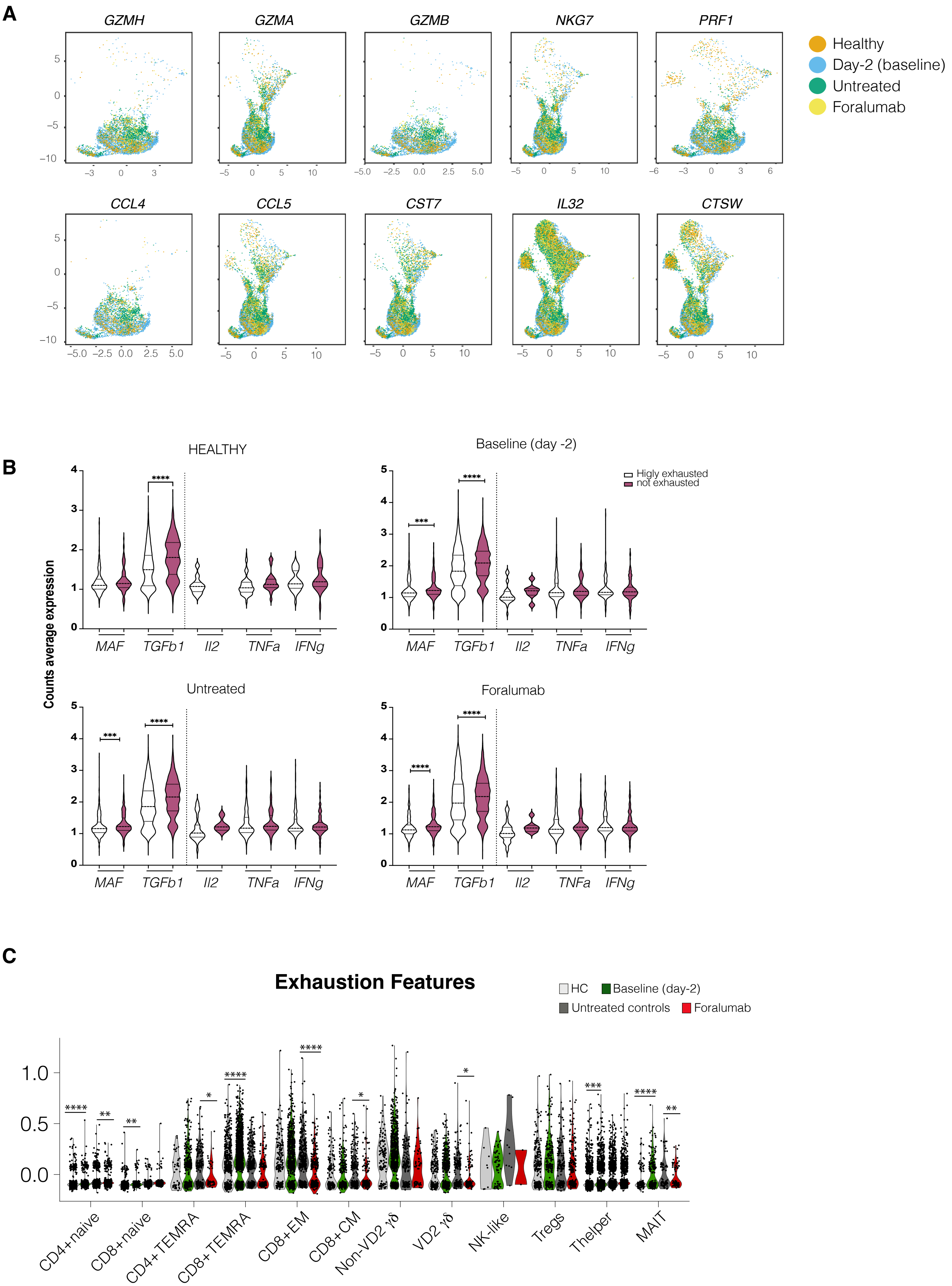
A
GZMH
GZMA
GZMB
NKG7
PRF1
CCL4
CCL5
CST7
IL32
CTSW
Healthy
Day-2 (baseline)
Untreated
Foralumab
B
HEALTHY
Baseline (day -2)
Untreated
Foralumab
Higly exhausted
not exhausted
Counts average expression
MAF
TGFb1
Il2
TNFa
IFNg
C
Exhaustion Features
HC
Baseline (day-2)
Untreated controls
Foralumab
CD4+naive
CD8+naive
CD4+TEMRA
CD8+TEMRA
CD8+EM
CD8+CM
Non-VD2 γδ
VD2 γδ
NK-like
Tregs
Thelper
MAIT

Figure S4. **Effector gene cluster and exhaustion modules in CD3+ T cells. A)** UMAP plots of T cells showing localization of effector genes in CD3+ cells across treatment. **B)** Exhausted score of >0.01 was used to determinate highly exhausted cells and compared to low/not exhausted cells (<0.01). *MAF, TGFB1, IL2, TNFa* and *IFNg* gene expression in exhausted and not exhausted cells is compared and shown in Violin-plots (Median±IQR). **C)** Distribution of exhaustion score within UMAP plots of CD3+ T cells subsets in in healthy controls, untreated COVID19 subjects and Foralumab treated subjects at baseline (day-2) and at day 10. Violin plots (Median±IQR) with individual points is shown. HC= healthy controls. One-way ANOVA, followed by Tukey *post hoc* analysis was performed and comparison between healthy vs. day-2 and untreated vs Foralumab is shown. * $P < 0.05$, ** $P < 0.01$, *** $p<0.001$, **** $P < 0.001$.

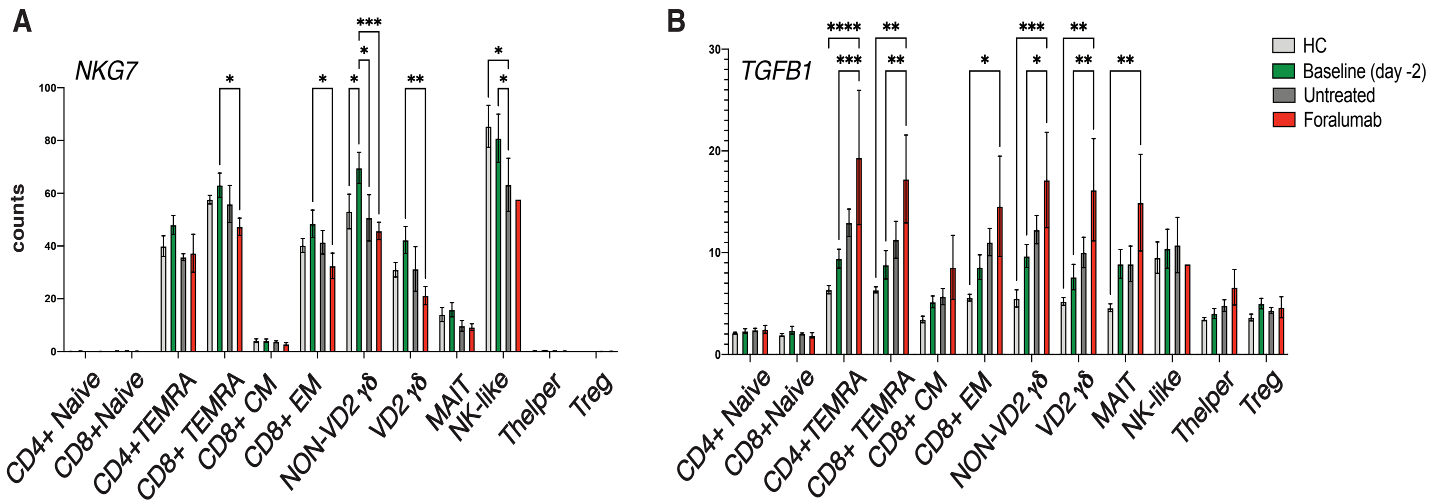

Figure S5. A, B) *NKG7* and *TGFB1* counts in CD3+ subsets in healthy controls, untreated and Foralumab treated COVID-19 subjects at baseline (day-2) and at day 10. *NKG7* **(A)** and *TGFB1* **(B)**. Bars represent mean±SEM. One-way ANOVA followed by Tukey *post hoc* analysis ** p<0.01. * P < 0.05, ** P < 0.01, **** P < 0.001.

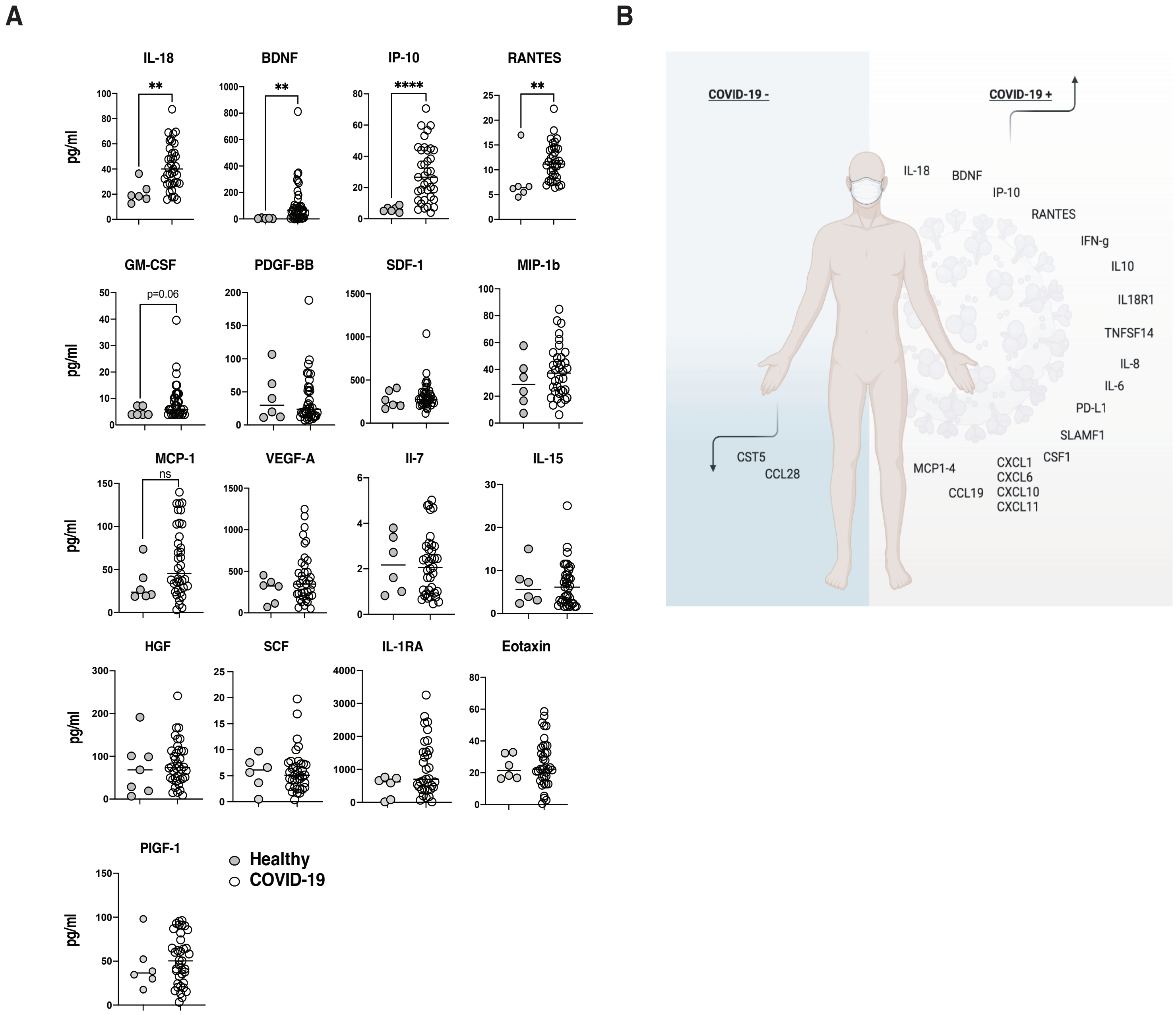

Figure S6. Serum markers altered by COVID-19 detected by proteomics. **A)** Comparison between healthy volunteers and COVID-19 subjects. Dots are individual values, bars represent mean±SEM. Student's t test. Healthy = healthy controls. COVID-19 refers to all COVID-19 subjects at baseline (day-2). **p < 0.01, **** P < 0.001. **B)** Resume of up and downregulated serum markers obtained by Olink.

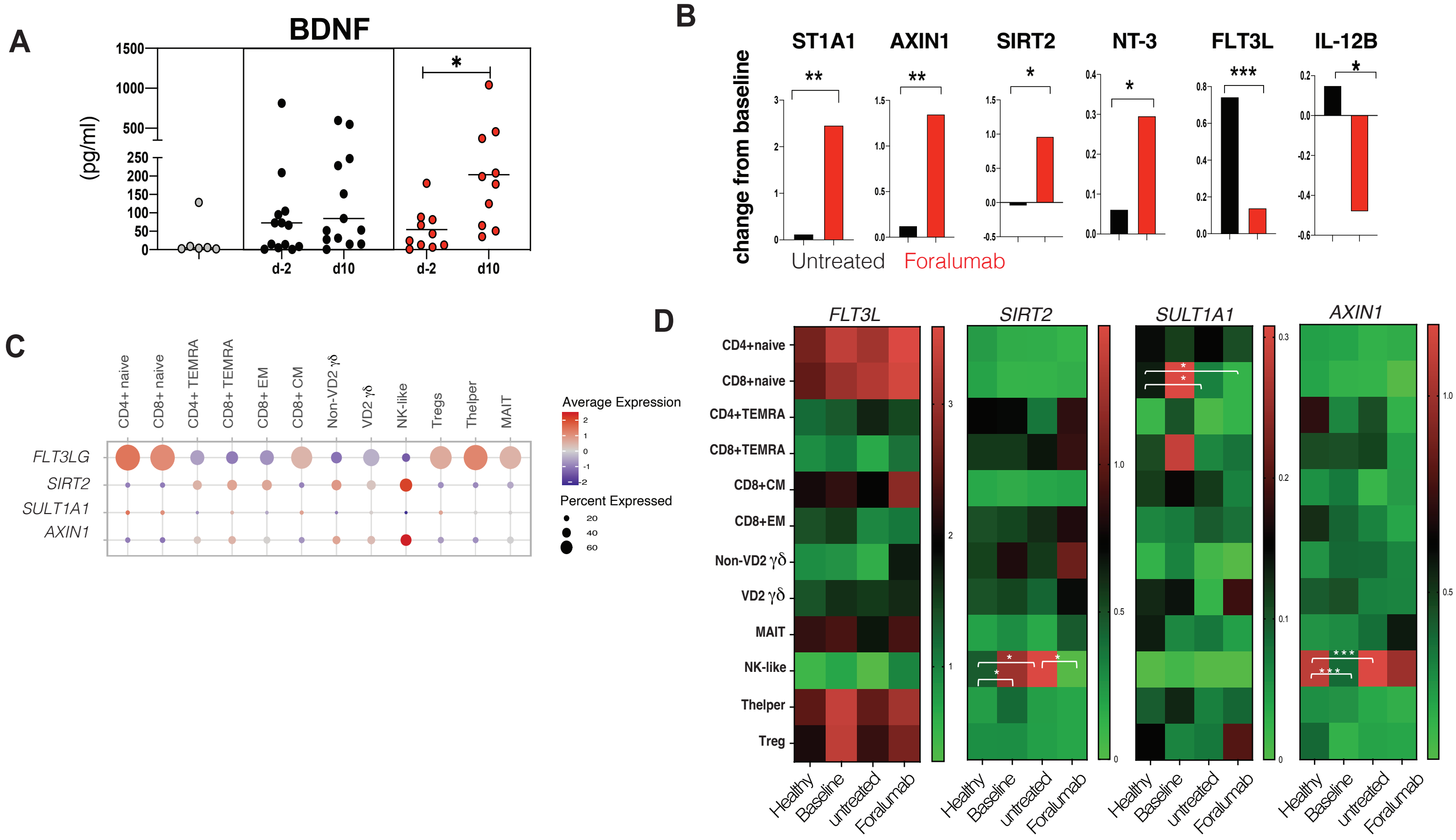

Figure S7. A) Serum BDNF before and after treatment measured by Multiplex. **B)** Serum ST1A1, AXIN1, SIRT2, NT-3, FLT3L and IL-12B change (before and after treatment) measured by Olink. Bars represent change from baseline. Student's t test. **C)** Dot plot showing average expression and percent of *FLT3G*, *SIRT2, SULT1A1* and *AXIN1* in $CD3^+$ cell subsets. **D)** Heat maps showing gene expression of the corresponding proteins found to be modulated by Foralumab treatment in sera. One-way ANOVA, followed by Tukey *post hoc* analysis was used for analysis in D. * $P < 0.05$, ** $P < 0.01$, ***$P = 0.001$

Table S1. Subject demographics

		Foralumab		Untreated		Healthy controls	
	Total	Gender (F/M)	Age (yrs)	Gender (F/M)	Age (yrs)	Gender (F/M)	Age (yrs)
Proteomics	n=33	12 (10/2)	44.5 ± 11	15 (9/6)	31 ± 18.9	6 (5/1)	42.4 ± 7
Bulk-RNA	n=23	8 (7/1)	47.6 ± 9.1	8 (6/2)	39.8 ± 19.1	7 (2/5)	31.9 ± 5.1
scRNAseq	n=12	4 (3/1)	46.7± 8.3	4 (3/1)	48.7 ± 24.5	4 (2/2)	35.6 ± 2.5

Proteomics: Multiplex and Olink
Bulk-RNA: Smart2-seq
scRNAseq: 10X genomics
Age ± Standard Deviation

Table S2: Serum Biomarkers before and after treatment measured by Multiplex

	Control					Foralumab				
	**Day -2**	**Day 10**				**Day -2**	**Day 10**			
	Mean (±SD) #		Change		p-value*	Mean (±SD)		change		p-value
IL.18	34.8 (±18.9)	32.2 (±17.7)	-2.6; 95% CI: -9.6, 4.3	- Ψ	0.429	46.9 (±15.5)	37.6 (±12.6)	-9.3; 95% CI: -18.9, 0.2	- Ψ	0.054
BDNF	144.4 (±217.6)	174.3 (±191)	29.9; 95% CI: -109.2, 169	-	0.651	91.5 (±105.6)	279.6 (±274.6)	188.1; 95% CI: 50.8, 325.4	↑	0.012
VEGF-A	455.6 (±391.8)	525.1 (±359.6)	69.5; 95% CI: -98.1, 237.2	-	0.389	362.1 (±184.7)	610.4 (±310.2)	248.3; 95% CI: 103.8, 392.9	↑	0.003
PIGF-1	49.2 (±29)	67.4 (±38.6)	18.1; 95% CI: -0.8, 37.1	-	0.059	55.8 (±28.7)	87.1 (±41)	31.3; 95% CI: 12.2, 50.5	↑	0.004
SCF	6.3 (±4.6)	6.8 (±3.7)	0.5; 95% CI: -0.6, 1.6	-	0.316	5.5 (±3.2)	6.6 (±4.1)	1.1; 95% CI: 0.2, 2	↑	0.023
HGF	73 (±43.9)	125.5 (±72.4)	52.5; 95% CI: 23.9, 81.1	↑	0.001	89.6 (±41.1)	187.2 (±89.5)	97.6; 95% CI: 63.7, 131.6	↑	<0.001
PDGF-BB	34.6 (±31)	77.5 (±78.4)	42.9; 95% CI: 0.3, 85.5	↑	0.048	48 (±49.5)	159.2 (±219.4)	111.1; 95% CI: -1, 223.3	-	0.052
IP-10	28.2 (±16.5)	12.3 (±8)	-15.9; 95% CI: -23.1, -8.7	↓	<0.001	30.9 (±20.2)	13.4 (±6.7)	-17.5; 95% CI: -29, -6	↓	0.006

Standard Deviation (SD)
*: P-value = difference within groups before (day-2) and after treatment (day 10) and controls. CI= confidence intervals
Ψ Increase (↑) Decrease (↓) No change (-)

IL= Interleukin; Brain Derived Neutrophic Factor (BNDF); Interferon gamma inducible protein-10 (IP-10); Placental Growth Factor (PIGF); Stem cell Factor (SCF); Hepatocyte Growth Factor (HGF); Vascular Endothelial Growth Factor A (VEGF-A); Platelet Derived Growth Factor- (PDGF).
